# INTERPLAY OF COGNITIVE STATUS AND SPOUSAL CAREGIVER PATTERNS ON WELL-BEING

**DOI:** 10.1093/geroni/igad104.1915

**Published:** 2023-12-21

**Authors:** Lindsay Ryan

**Affiliations:** University of Michigan, Ann Arbir, Michigan, United States

## Abstract

The current paper investigated the intersection of cognitive status and caregiver characteristics on older adults’ reports of life satisfaction and depressive symptoms. Using a sub-sample from the 2016 Health and Retirement Study Harmonized Cognitive Assessment Protocol (HRS-HCAP), individuals were selected if the HCAP informant was a spouse self-identified caregiver. The sample was trimmed to include those whose spouse caregiver was also a respondent in the main 2016 HRS and completed the measures of interest in the psychosocial leave-behind questionnaire (N = 190). The final analytic sample comprised 109 respondents classified as cognitively normal, 51 with cognitive impairment, not dementia, and 30 with dementia based on the algorithm developed by Manly, Jones, Langa, et al. (2022). The sample was 50% women, 10% racialized as Black, 18% racialized as LatinX, with a mean age of 75.62 and an average 11.75 years of education. Latent Class Analysis identified meaningful caregiver psychosocial classes from spouse reports on purpose in life (Ryff & Keyes, 1995), positive and negative affect (Watson & Clark, 1994), and perceived positive and negative support from one’s spouse (in this case the main respondent), children, other family, and friends (Turner, Frankel, & Levin, 1983). The data supported a 4-class solution, which was then used in linear regression analyses to examine associations of cognitive status and caregiver profiles on individual’s life satisfaction and depressive symptoms. Interestingly, cognitive status was not associated with well-being in the current study, suggesting associations of spousal psychosocial classes were independent of the care-recipient’s dementia/impairment designation.

